# Haptic Feedback Device Using 3D-Printed Flexible, Multilayered Piezoelectric Coating for In-Car Touchscreen Interface

**DOI:** 10.3390/mi14081553

**Published:** 2023-08-02

**Authors:** Van-Cuong Nguyen, Victor Oliva-Torres, Sophie Bernadet, Guilhem Rival, Claude Richard, Jean-Fabien Capsal, Pierre-Jean Cottinet, Minh-Quyen Le

**Affiliations:** 1 LGEF, INSA-Lyon, EA682, University Lyon, 69621 Villeurbanne, France; van-cuong.nguyen@insa-lyon.fr (V.-C.N.); victor.oliva-torres@insa-lyon.fr (V.O.-T.); guilhem.rival@insa-lyon.fr (G.R.); claude.richard@insa-lyon.fr (C.R.); jean-fabien.capsal@insa-lyon.fr (J.-F.C.); 2 Arc en Ciel Sérigraphie, Z.I Le Forestier, 42630 Regny, France; sbernadet.ingenieur@arcenciel-serigraphie.fr

**Keywords:** haptic feedback device, 3D screen printing, flexible smart coating, design optimization, piezoelectric actuator/sensor, finite element simulation

## Abstract

This study focuses on the development of a piezoelectric device capable of generating feedback vibrations to the user who manipulates it. The objective here is to explore the possibility of developing a haptic system that can replace physical buttons on the tactile screen of in-car systems. The interaction between the user and the developed device allows completing the feedback loop, where the user’s action generates an input signal that is translated and outputted by the device, and then detected and interpreted by the user’s haptic sensors and brain. An FEM (finite element model) via ANSYS multiphysics software was implemented to optimize the haptic performance of the wafer structure consisting of a BaTiO_3_ multilayered piezocomposite coated on a PET transparent flexible substrate. Several parameters relating to the geometric and mechanical properties of the wafer, together with those of the electrodes, are demonstrated to have significant impact on the actuation ability of the haptic device. To achieve the desired vibration effect on the human skin, the haptic system must be able to drive displacement beyond the detection threshold (~2 µm) at a frequency range of 100–700 Hz. The most optimized actuation ability is obtained when the ratio of the dimension (radius and thickness) between the piezoelectric coating and the substrate layer is equal to ~0.6. Regarding the simulation results, it is revealed that the presence of the conductive electrodes provokes a decrease in the displacement by approximately 25–30%, as the wafer structure becomes stiffer. To ensure the minimum displacement generated by the haptic device above 2 µm, the piezoelectric coating is screen-printed by two stacked layers, electrically connected in parallel. This architecture is expected to boost the displacement amplitude under the same electric field (denoted E) subjected to the single-layered coating. Accordingly, multilayered design seems to be a good alternative to enhance the haptic performance while keeping moderate values of E so as to prevent any undesired electrical breakdown of the coating. Practical characterizations confirmed that E=20 V/μm is sufficient to generate feedback vibrations (under a maximum input load of 5 N) perceived by the fingertip. This result confirms the reliability of the proposed haptic device, despite discrepancies between the predicted theory and the real measurements. Lastly, a demonstrator comprising piezoelectric buttons together with electronic command and conditioning circuits are successfully developed, offering an efficient way to create multiple sensations for the user. On the basis of empirical data acquired from several trials conducted on 20 subjects, statistical analyses together with relevant numerical indicators were implemented to better assess the performance of the developed haptic device.

## 1. Introduction

### 1.1. Principle of Haptic Feedback System and Objective of This Work

Today, human interaction with technology mainly focuses on the visual and auditory aspects of perception, the most prominent senses that humans rely on for interpreting the world around them [1,2]. However, the complete human experience is not limited to these two, but formed from five basic senses (sight, hearing, touch, smell, and taste) [3,4]. While the incorporation of smell and taste into technology interfaces is still in the early stages of development [5], the sense of touch is becoming an increasingly important component of human interaction with technology [6,7,8]. By introducing proper implementation, modern devices enable users to not only see or hear, but also literally feel and experience virtual environments and interactions more realistically [9,10,11]. This is exactly where haptic feedback comes into play.

Haptic feedback relies on two systems to engage the haptic sense—one on the human side and one on the machine side. The human side includes the haptic sensors in the skin and the neural pathways that transmit information to the brain. The machine side includes the sensors that detect the user’s touch, the algorithms that interpret the input, and the actuators that generate the haptic feedback [12]. The process of haptic feedback involves the following steps:

- The user interacts with a haptic-enabled device such as a touchscreen by applying pressure or making contact with the surface. This action generates an input signal detected by the machine’s sensors.
- The signal is translated by the machine’s algorithms into a haptic response, such as a vibration or force.
- This response is then outputted by the machine’s actuators, which generate the physical sensation perceived by the user.
- The user’s haptic sensors in the skin detect the physical sensation and transmit information to the brain, which interprets the sensation as a particular texture, shape, or force.

The objective here is to explore the possibility of developing a haptic interface to replace mechanical buttons thanks to piezoelectric materials; hence, vibrotactile feedback is the technology used in our target application. In order to build an effective haptic feedback demonstrator, it is important to have a good understanding of how humans perceive touch, the sensitivity of human skin, and the limitations of human sense of touch. Indeed, our skin is only able to detect a certain range of frequencies and amplitudes, and this range can vary depending on the location on the body [13]. Additionally, different types of receptors in the skin are sensitive to different types of stimuli, such as pressure, temperature, and vibration. Understanding these factors allows designing haptic feedback systems that are optimized for specific applications and provide a more realistic and immersive user experience.

Some researchers have tried to describe the absolute perception threshold [14,15], when humans start to perceive an interaction in their skin. Haptic perception was demonstrated to be dependent on various numbers of factors such as force, the positioning of the body part, displacement, and frequency [16]. According to the perception threshold curve characterizing the penetration depth as a function of the frequency [17,18,19], the maximum sensitivity has been identified at around 300 Hz (i.e., corresponding to a minimum penetration depth of 2 µm). Therefore, to maximize the perception of the user, this study involves the development of a haptic system capable of generating a displacement of at least 2 µm within a bandwidth of 100–700 Hz. It is worth noting that such characteristics are validated with large transmission areas (>$1\;cm^{2}$) of input force, similar to a large button used in a tactile screen. In addition, the piezoelectric button must be capable of withstanding a maximum force of 5 N generated by the fingertip. 

To reach the full performance of haptic functionality, the developed device must fulfill the following criteria: mechanically flexible, adaptable to different sizes and shapes, cost-effective, easy process, and low environmental impact. In that situation, the printing technique becomes a viable option for achieving the deposition of multilayered materials in additive manufacturing (AM) that are of various sizes and shapes [20]. In particular, among the various methods currently in use [21,22,23,24,25,26,27], screen printing appears to be a quick, effective, straightforward, durable, and inexpensive process that may be used on an industrial scale [28,29,30,31]. In our case, the conception of a flat substrate with multiple layers of different materials has been revealed to be perfectly suitable for AM using screen printing. 

Regarding the process, the piezoelectric ink was screen-printed on a thin and flexible substrate made of polyethylene terephthalate (called PET) thermoplastic polymer resin. This polymer has the advantage of being colorless, flexible, low-cost, and commercially available, with an easy process and good adhesion with a variety of inks [32,33,34,35,36]. Several layers of piezoelectric coating are conducted to produce sufficient vibration that can be felt by the user’s fingertip. The haptic button is designed as a wafer structure made of lead-free barium titanate (BaTiO_3_) composite, sandwiched between two silver electrodes, and a substrate layer of a flexible PET substrate. When the button is pressed, the force applied to the piezoelectric wafer is measured via a load cell and generates a voltage (sensor mode) detected by a specific algorithm. This detection allows for the activation of a high voltage applied to the wafer, which in turn generates a dynamic mechanical strain exerted on the button (actuator mode) [37]. Finally, the resulting strain creates a feedback sensation that can be felt by the user, similar to the feeling of pressing a mechanical button. The direct and inverse effects of the piezoelectric material make the design of haptic buttons quite ergonomic, compact, and multifunctional (i.e., able to perform in both sensor and actuator configurations). In addition, combining such a smart material design with 3D printing AM leads to an easier processing of multilayered architecture and overcomes critical issues imposed by the design rules of an electrical circuit [38]. 

The exploration of this study is of high interest, especially in the automobile field, where the touchscreen is mandatory for the interaction with the driver. As the user interfaces of in-car systems become more sophisticated, controlling them hands-free without looking directly at the screen becomes challenging. The lack of tactile feedback causes the user’s brain to look at the screen to confirm input selection [39]. By adding the haptic feedback to the touch displays, the on-board computer can confirm the driver’s inputs through variable vibrations, confirming whether or not their action was correctly recorded [40]. A haptic demonstrator is successfully developed at the end of this study which satisfies the technical specifications imposed by the automotive sector.

### 1.2. Materials Used for Haptic Touchscreen Actuators 

Haptics is found in nearly all modern touchscreen devices to increase input/typing accuracy while subsequently improving user satisfaction. Haptic technologies use a variety of methods (resistive [41], capacitive [42,43], surface acoustic wave (SAW) [44], infrared grid [45], etc.) to create the sense of touch. Table 1 summarizes different types of actuators for generating tactile stimuli on touch surfaces together with their advantages and inconveniences. 

The electromagnetic actuators generate a force that is proportional to a current. Among others, vibrations motors, consisting of a DC motor that has a rotor with eccentric mass distribution (so-called ERM—eccentric rotating mass), have been used most widely for surface haptic applications [46,47,48,49]. ERM creates rotational movement in the housing that is perceived as a vibration by the user. It can generate relatively large vibrations and be made in diverse sizes and shapes [50]. The frequency of vibration is controllable by changing the voltage. However, the amplitude (in displacement) is constant, which restricts the diversity of vibration waveforms that can be rendered. Furthermore, they generally have large actuation lags with low dynamics. 

Linear resonance actuators (LRAs) are a special voice coil actuators designed in a small size for mobile devices. While they have a faster response than an ERM, their frequency bandwidth is generally very narrow. In general, electromagnetic actuators are usually suitable for low- to mid-range actuation frequencies (with respect to human tactile perception), have low voltage requirements, and have high displacement/low force characteristics [51]. 

Electrostatic actuators involve applying a voltage to the conductive layer of a capacitive touchscreen to generate electrostatic attractive forces in the normal direction between its surface and a finger sliding on it [52], which leads to an increase in frictional forces applied to the finger opposite to the direction of movement. The level of force, which is proportional to the square of the voltage, is typically extremely low. Nonetheless, electrostatic actuators can be used for high-dynamics haptic applications, allowing to overcome the main drawback of the electromagnetic technology.

Piezoelectric ceramic actuators, excellent alternatives for actuation at high frequencies, are also frequently used for surface haptics [53,54,55]. In addition, they are compact, with high electromechanical coupling, adaptable for robocasting 3D printing technology [56], and suitable to drive high output force. However, they generate low displacement, have a complex process and integration, and are brittle and weak to external shock, which are considered major drawbacks preventing their commercial adoption.

Electroactive polymers have been revealed as very attractive for providing haptic feedback in touchscreen devices as they can be miniaturized, are durable, and have large active strains in response to driving voltages, along with high power density, good mechanical compliance, and structural simplicity; most importantly, they offer efficient and versatile actuation for effective user interactions [57,58,59,60]. Novel inorganic ferroelectrics, such as lead-free Bi_0.5_Na_0.5_TiO_3_ (BNT) and antimony sulfoiodide nanowires, also exhibit remarkable piezoelectric properties. However, their need for a high sintering temperature is not compatible with most flexible substrate materials [61,62] that are essential in most applications of haptic devices. Polyvinylidene fluoride (PVDF) and its co- and ter-polymers can be seen as some of the best candidates for mechanical and acoustic sensors, actuators, and energy harvesters [63,64,65]. Ju et al. demonstrated tactile feedback on flexible touch screens using transparent relaxor ferroelectric polymer film vibrators with solution-processed but nonprinted poly(vinylidene fluoride–trifluoroethylene–chlorotrifluoroethylene) (P(VDF–TrFE–CTFE)) as the active layer. For a cost-effective production and the realization of large-area flexible actuators, the combination of organic actuator materials and additive printing technologies can play an important role. Poncet et al. presented the study of screen-printed P(VDF–TrFE)-based haptic circular buttons, providing force restitution or vibration sensations when touched by the user, causing a tactile sensation on the human fingertips [66]. Nonetheless, very limited experimental characterization was provided to confirm the actuation performance of the proposed device, particularly the behavior of the displacement/vibration as a function of the input force applied by the fingertips. Schmidt et al. developed a new design of printed multilayer piezoelectric transducers for haptic feedback, also using P(VDF–TrFE) copolymer [67]. It was pointed out that the transducer could generate a maximum blocking force of approximately 0.6 N at the resonance frequency of 320 Hz, which seems to be insufficient for the haptic feedback device. In general, electroactive polymers, with their excellent softness, are easily prepared and implemented on a large surface in various sizes and forms. However, they usually generate low output force and require high-voltage application, and they are particularly expensive compared to the former techniques based on electromagnetic and electrostatic actuators.

In this paper, we propose another class of materials—piezoelectric composites—that combine the benefits of both piezoelectric ceramic fillers and dielectric polymer. As indicated in Table 1, the piezocomposites make it possible to achieve the best compromise between mechanical flexibility and the actuation responses (force and displacement) in terms of amplitude and frequency. Furthermore, such materials are cost-effective, easily processed, and suitable for 3D printing AM without adhesion treatment of the substrate; they have moderate voltage requirements and the possibility to function in sensor mode. To the best of our knowledges, piezoelectric composites have not yet been widely explored in haptic tactile devices. Actually, the performance of these systems strongly depends on several parameters of the fillers (e.g., nature, size, shape, dispersion, and concentration), of the matrix (e.g., dimension, mechanical flexibility, temperature stability, adhesion with substrate, and viscosity), and of the interconnectivity between them (i.e., arrangement of the fillers within the matrix) [68,69,70,71,72,73,74]. To some extent, these features give multiple possibilities to improve the system performance through adjustments of the materials’ parameters. However, full characterizations are usually required to have a clear understanding of about many factors, ultimately related to each other, that cannot be decoupled in certain circumstances. 

Last but not least, recent advances in smart and multifunctional materials have been actively explored among the scientific community in the area of tactile touchscreen, but a lack of understanding of their characteristics (e.g., displacement versus force, frequency-dependent characteristics of actuation, influence of geometric parameters, etc.) somewhat limits their wide use in real systems. Although the importance of the haptic feedback is generally realized, there is a limited number of formal studies on how to optimize the design of haptic feedback on a touchscreen, and how the satisfaction of different haptic waveforms is perceived. On the basis of these critical issues, the new insight of this study involves the development of haptic functions using a multilayered piezoelectric composite coating that can perform in both sensor and actuator modes. A progressive optimization design of the system was developed using the finite element model (FEM) of ANSYS software (student version 23.1.0.0), demonstrating the impact of relevant parameters on the haptic performance. Full characterizations of actuation ability as a function of the frequency, the input user’s force, and the applied electric field were carried out through practical tests. The results are expected to determine an adequate level of electric field, moderate but sufficient enough to activate the vibrations sent back to the user. The displacement driven by the device must be beyond the detection threshold (~2 µm, minimum level that can be perceived by the user) regardless of the input force level (~maximum 5 N). To increase the interaction with the user, an interesting approach involves the combination of tactile and audible sensation. In addition to vibration measurements, Schmidt et al. performed acoustical sound pressure level (SPL) measurements in the audible frequency range from 100 Hz to 20 kHz, at a distance of 10 cm [67]. It was pointed out that a remarkably high SPL of more than 90 dB was achieved for a wide frequency range from ~2 kHz to 17 kHz. In the haptic applications where the low-frequency range between 100 and 500 Hz is important, the SPL is supposed to be much lower, which is actually beneficial as the audible noise is comparably small. In this study, we investigate statistical analyses from practical trials conducted on several subjects. Each subject had to express their level of satisfaction (from 0% to 100%) with the haptic sensation and the acoustical noise. 

## 2. Materials and Process

### 2.1. Material Selection and Ink Formulation

To develop the piezoelectric ink, polyurethane acrylate (PUA), one of most popular photocurable resins based on the thermoplastic PU, was chosen as the polymer matrix. PUA has attracted much attention in ultraviolet (UV)-curable coatings attributed to its excellent flexibility, prominent adhesion on substrates, and variety of adjustable features, ensuring adhesion on the PET substrate [75,76]. In this study, the piezoelectric ink, developed according to a collaboration between LGEF and VFP Ink Technologies, should fulfill the following specifications:

- Compatible with 3D printing additive manufacturing (AM): easy manipulation (adequate viscosity and dispersion) and good adhesion with the PET substrate.
- Durable: ability to withstand handling, heat, moisture, and other environmental factors.
- Cost-effective materials and printing process.

**Figure 1:**
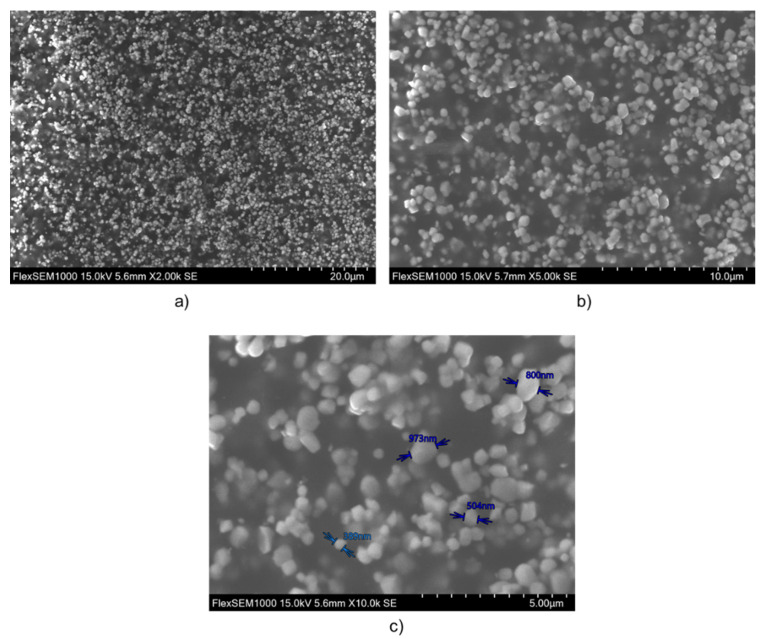
SEM image of BaTiO_3_/PUA composite with different magnifications: (**a**) ×2k; (**b**) ×5k; (**c**) ×10k.

The ink was formulated through the functionalization of barium titanate (BaTiO_3_) spherical nano-powder, with the aim of preventing agglomerations when suspended in the ink matrix [77,78]. The nano-powder BaTiO_3_ was purchased from Inframat^00^ Advanced Materials TM (Product # 5622-ON7), with a purity of 99.95 % and a density of 5.85 $g.{cm}^{-3}$. As observed by scanning electron microscopy (the SEM) via a Hitachi Flex SEM 1000II microscope), the particles are quasi-spherical (Figure 1), with an average size of around 600–700 nm. These particle sizes seem to be the best compromise between material dispersion and performance in electromechanical conversion [23]. Indeed, the composite piezoelectric activity decreases for particles size lower than 300 nm due to loss of the tetragonal phase [79].

### 2.2. Fabrication Process Based Screen-Printing Technique

The additive manufacturing (AM) of multilayered piezoelectric composite coated on a PET transparent substrate was conducted via an industrial process piloted by ACE (Arc en Ciel Sérigraphie). For more details about the process, interested readers can refer to [76]. BaTiO_3_/PUA composite ink was employed for the piezoelectric coating, reticulated under a UV light (405 nm wavelength), with an irradiance power of 300 $mJ/cm^{2}$. The silver ink was used for the electrodes and conductive tracks (CTs), and then thermally cured in a drying oven (SIEBDRUCK TRO II) between 100 and 150 °C. It has been revealed that the screen printing allows forming a very thin film coating, on the order of 25–30 µm, leading to an easier polarization process and reduced breakdown probability [80,81]. In addition, the proposed technique makes it easy to create multilayered structures by repeating the process as in the case of the single layer. 

**Figure 2:**
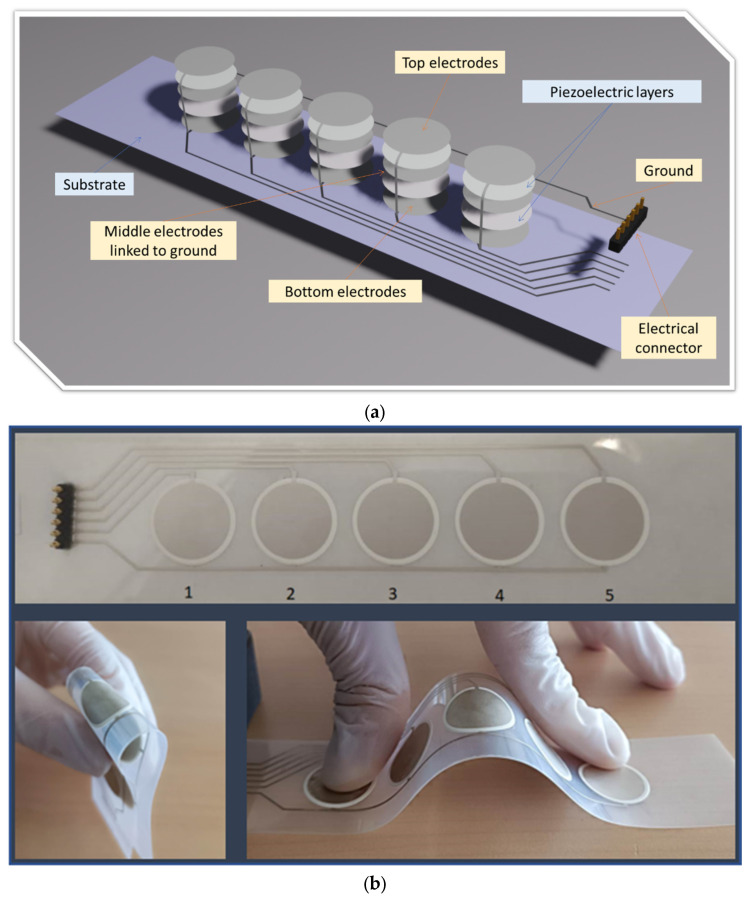
From design to real prototype: (**a**) architecture of two-layer piezoelectric actuator; (**b**) real prototype of piezoelectric actuator coating printed on a flexible PET substrate.

Figure 2a shows an example of five multilayered piezoelectric elements, where each is considered as two capacitors in parallel thanks to the connection of three electrodes. The five middle electrodes are all linked to the electrical ground via the lower CT (on the right-hand side). The circular top and bottom electrodes of each active element are interconnected among themselves and linked to one of the five connector pins via an upper CT (on the left-hand side). The final customized product is shown in Figure 2b, including five identical circular piezoelectric elements numbered 1–5. A six-pin electrical connector is implemented on the extremity of the dielectric PET layer, linking to the conductive tracks. The distance between two successive pins is large enough (~2.54 mm) to avoid any electrical arc in case of high-voltage applications. The design of the CTs must fulfill the critical rules descried previously in [38] so as to reduce parasitic capacitance throughout empirical measurements. Noted that the printed sample is flexible enough to be foldable and stretchable, which is perfectly adequate in applications of haptic touchscreens.

### 2.3. Poling Procedure

The poling setup consists of a waveform generator (Agilent 33210A, Keysight Technologies Inc., Santa Rosa, CA, USA) coupled to an amplifier (10/10 B-HS, TREK Inc., Novi, MI, USA) by a factor of 1000. The DC electric field (*E*) was progressively increased until the desired value and kept constant for a few minutes. For polarization at high temperature, the entire sample holder was placed into an oven (Votsch Industrietechnik TM, VT-7004, Bergen, Germany), allowing the control of temperature with high precision. Afterward, samples were progressively cooled down at room temperature under a magnetic field. As soon as the field was removed, samples were short-circuited during 5 min to totally evacuate undesired electrostatic charge. To check the success of the polarization procedure, the piezoelectric sensitivity (*d*_33_, usually defined as the longitudinal charge coefficient) was measured through a piezometer (Ye2730a–D_33_ Meter, Global Sensor Technology, Manchester, UK).

## 3. Characterization Methods

### 3.1. Setups for Characterization of Haptic Performance

**Figure 3:**
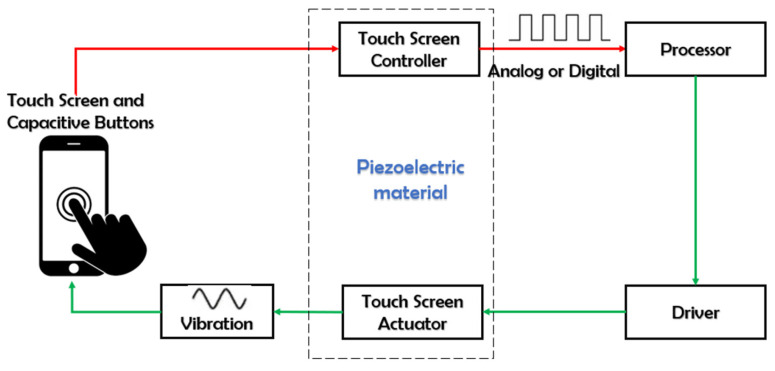
Working principle of a haptic touchscreen system using flexible printed piezocomposite.

These experimental setups aim to evaluate the performance of the piezoelectric material as a haptic system, by measuring its ability to convert electrical energy into mechanical motion (actuator mode) that would be felt by user. Furthermore, the piezoelectric element is supposed to be tested in the sensor mode when the user performs a touch command that can be detected by the piezoelectric device. Consequently, the system is activated by producing vibration feedback, a so-called touch sensation. Figure 3 illustrates a typical haptic system composed of a touch device with a capacitive button, processor, drive circuit, and actuator. The input of the haptic system might be a touch consisting of a capacitive button that serves as an input to a touchscreen device. It detects touch pressure that is sent to the processor in the form of an analog or digital signal. The processor generates a specific waveform according to the touch; finally, the piezoelectric actuator creates movements on the basis of the waveform conducted by the processor.

The test procedure of the piezoelectric haptic feedback device can be split into the following steps [82]:

- Preparation: The piezoelectric actuator is mounted in a test setup, including a power supply, measurement instrument, and any necessary fixtures or support structures.
- Mechanical-to-electrical sensing: A user applies a mechanical force or displacement to the piezoelectric material that results in the generation of an electrical signal, such as voltage or charge (corresponding to red arrows of Figure 3).
- Electrical-to-mechanical actuation: After receiving the trigger mentioned above, an electrical voltage is driven to the piezoelectric element, producing a mechanical motion (such as displacement, strain, or force) that is detected by the user’s fingers (corresponding to green arrows of Figure 3).
- Characterization: The results of the electrical-to-mechanical (actuator) and mechanical-to-electrical (sensor) tests are analyzed to determine the piezoelectric response and validate the haptic performance, such as frequency response, amplitude level, and stability.
- Comparison: The results are compared to the desired specifications and tolerances to determine if the actuator meets the performance criteria of a haptic touchscreen device.

#### 3.1.1. Mechanical-to-Electrical Test Setup

Our objective in the sensor mode is to detect the user input when the button is pressed, and to determine when and how a haptic response is sent to the user. The pressing force applied on the button is measured using a force-sensing resistor, known as “FlexiForce”, whose resistance changes with the input force [31]. A calibration of FlexiForce was conducted via a Sirius 8XSGT card interfaced with the DEWE software (DewesoftX 2022.4). This led to the following relationship between the force (*F*) and the variable resistor ($R_{ca})$:

(1) $$F=67.4R_{ca}^{-0.784}.$$

$R_{ca}$ can be determined from the measurement of output voltage (*V_s_*) using a voltage divider circuit:

(2) $$R_{ca}=\frac{150V_{s}}{\left(1-\frac{V_{s}}{1000}\right)}.$$

Equations (1) and (2) were set into the DEWE-Sirius device, allowing to determine the force applied by the user’s fingertip. 

#### 3.1.2. Electrical-to-Mechanical Test Setup

**Figure 4:**
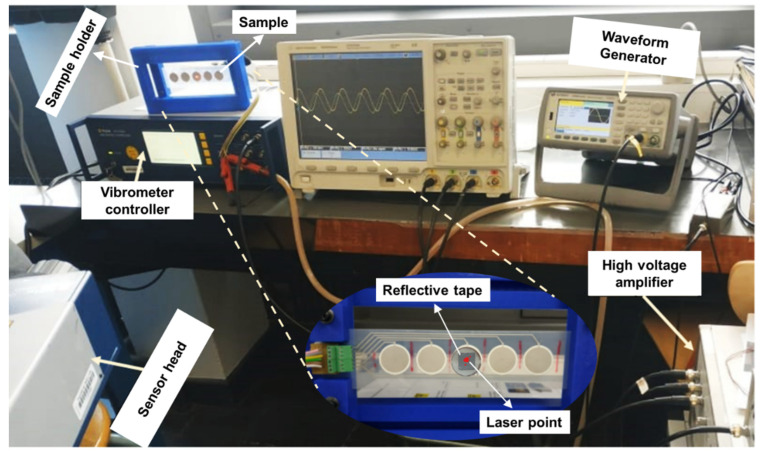
Setup opted for in the electrical-to-mechanical actuation test without force measurement. The inset image at the bottom is a zoomed-in view of the sample and its support.

The test setup for the electrical-to-mechanical actuation is illustrated in Figure 4. This setup includes five main elements: the piezoelectric actuator specimen, a waveform generator (Agilent 33220A), a high-voltage amplifier (TREK 10/10B-H-CE-EX), and a vibrometer controller (OFV-5000-2G) with a sensor head (OFV-505). Typically, mechanical vibrations captured by the sensor of a vibrometer are amplified and processed through a specified control unit, and then converted into an exploitable output signal. The sensor head emits a laser beam pointed to the piezoelectric actuator via a reflective tape glued on its surface (see Figure 4 for a zoomed-in view on the sample). As a result, the displacement of the sensor is locally measured with a high resolution. A voltage signal, delivered by a sinusoidal waveform generator with tunable amplitude and frequency, was amplified and driven to the piezoelectric specimen, which in turn induced its displacement. The signals delivered from the vibrometer and the waveform generator are monitored and recorded using an oscilloscope (IDS-1054B) or a Sirius 8XSGT card interfaced with the DEWESOFT software application (DewesoftX 2022.4).

On the basis of the velocity magnitude ($v_{max})$ of the piezoelectric specimen measured with the vibrometer, it is possible to infer the maximum displacement ($d_{max}$) according to the following expression:

(3) $$d_{max}=\int_{0}^{\frac{T}{4}}v_{max}\sin(\omega t)dt=\frac{v_{max}}{2\pi f},$$

where T, ω, and f are respectively the period, the angular velocity, and the frequency of the induced velocity signal, which is supposed to be sinusoidal (i.e., similar to the applied voltage) regarding the linear piezoelectric response. 

As $d_{max}$ is frequency dependence, the selection of an adequate operating frequency is of primary importance to activate enough motion to the haptic system. Moreover, the frequency also has an impact on the human’s perception, which is in turn affected by the displacement level of the tactile feedback. After several empirical trials, it was pointed out that, with a displacement of a few micrometers activated by the piezoelectric thin-film actuator, the set frequency of around hundreds Hz enables users to clearly perceive vibrations generated to their finger skin. 

Knowing $d_{max}$, the maximum deformation (or the strain, denoted as $S_{3\_max}$) along the thickness direction (i.e., 3-axis) of the piezoelectric actuator can be inferred as

(4) $$S_{3\_max}=\frac{d_{max}}{e},$$

where e is the thickness of the piezoelectric element. Under a given electric field (denoted $E_{3}$) applied along the three axes, the induced strain $\left(S_{3}\right)$ ultimately depends on the property of the piezoelectric material (i.e., the charge coefficient $d_{33}$) according to the following expression:

(5) $$S_{3}=d_{33}E_{3}.$$

The above relationship was carried out under load-free conditions. As indicated in Equations (4) and (5), for a given input voltage, a sample with higher thickness leads to higher displacement, giving rise to enhanced actuation performance. As a matter of fact, several piezoelectric layers were stacked together to create a multilayer structure so as to improve human perception.

**Figure 5:**
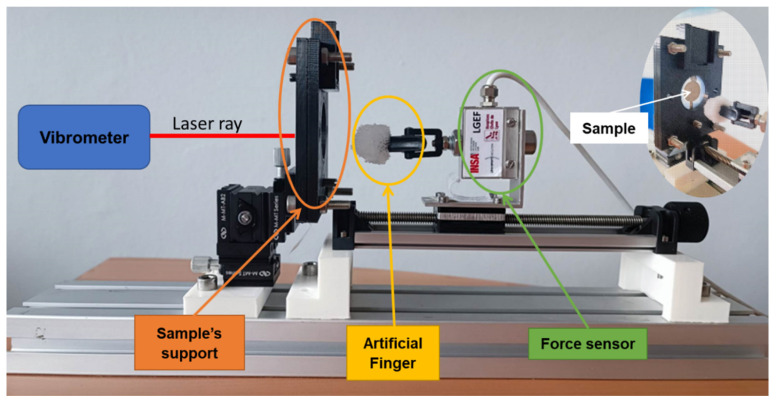
Setup opted for in the characterization of electrical-to-mechanical actuation with the force measurement. The inset image in the top right conner shows the front view of a piezoelectric sensor with its support.

Figure 5 illustrates an experimental setup that enables measurement of the force applied to the piezoelectric specimen. For the sake of simplicity, the sample was made of one circular coating (with 18 mm diameter and 28 µm thickness), which was clamped by a homemade square support (with a side length of 85 mm). One side of the piezoelectric sample was bonded with a reflective tape on which a laser ray issued from the sensor head of the vibrometer was pointed. The other side, simulated as a button on a tactile screen, was pressed by an artificial finger (see Figure 5) that could be slid along the translation support. The customized finger was made of plastic whose elasticity was similar to that of a human’s finger. 

The experiment started when the artificial finger presses the piezoelectric button capable of withstanding a force of a few newtons (i.e., not exceeding 5 N). The finger was manually piloted by the user along the translation direction thanks to a micrometer-screw system, leading to highly precise and smooth movements. A load sensor (DOERLER Measures LC 102 TC) attached to the finger allowed for the determination of the applied force. The force level depended on the displacement of the finger when making contact with the piezoelectric specimen. As soon as the contact is occurred, the sample was stressed and, thus, delivered an electrical signal that was detected by an algorithm developed using Digilent Analog Discovery 2 (sensing effect). An input voltage was activated to apply to the sample, which in turn generated a vibration that can be perceived by the finger (actuating effect). As the sensation of touch via the artificial finger was not obvious, a record of the motion through the vibrometer was carried out to assess the reliability of the haptic device. Through the determination of the piezoelectric displacement, it was possible to confirm whether or not such a movement could be sensed by the human’s finger. For an easier observation, the input voltage was tuned with a constant frequency of 300 Hz and an amplitude of 560 V (i.e., equivalent to an electric field of 20 V/µm) to create a stable deformation over time. Both force and motion signals were simultaneously monitored and recorded using a DEWE card interfaced with a computer via DEWESOFT.

### 3.2. Simulation Model Used for Design Optimization

**Figure 6:**
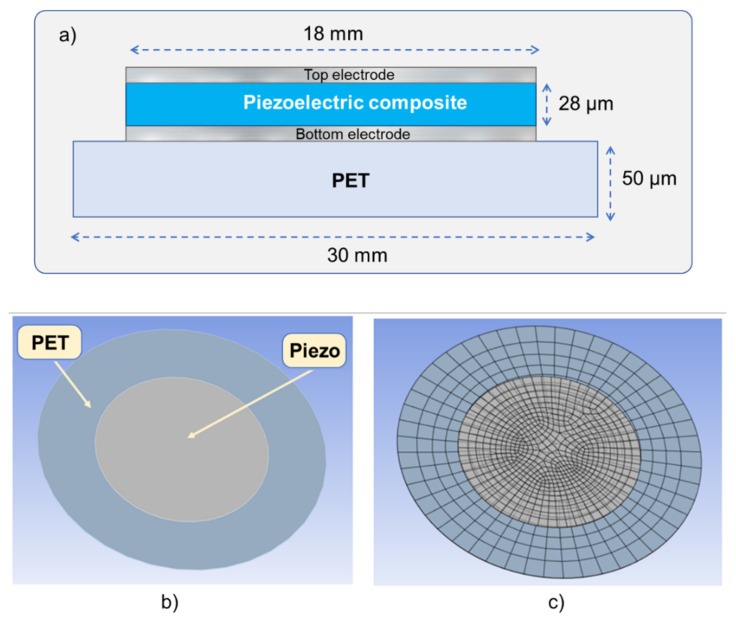
(**a**) Geometry of a simple piezoelectric actuator; (**b**) FEM of piezoelectric actuator built on ANSYS software; (**c**) mesh pattern used in simulation.

To optimize the design of a piezoelectric actuator, a finite element model (FEM) built on ANSYS simulation software was investigated. As illustrated in Figure 6a, a simple geometric design of the piezoelectric actuator consisted of a piezocomposite layer sandwiched between the top and the bottom electrodes. They were all stacked together above a circular PET substrate that was fixed along its circumference. The diameter and the thickness of these layers, indicated in Figure 6a, could be modified to optimize efficiency of the actuator. From the practical point of view, electrodes are indispensable to make electrical connections throughout a measurement, which is usually needless in simulation model. However, the presence of electrodes has an impact on the mechanical flexibility, which in turn might affect the actuation performance of piezoelectric devices. To better address this issue, a full piezoelectric model combined with two thin electrodes (~10 µm) was considered in the FEM model (see Figure 6b). For the best balance between the computing time and the model accuracy, the mesh size of the PET substrate was significantly larger than that of the piezoelectric layer (see Figure 6c). The properties of the entire specimen used in FEM are shown in Table 2, which relied on the following assumptions, for the sake of simplicity:

- The piezoelectric layer is built from isotropic materials, meaning that electrical and mechanical properties are directionally independent.
- The conductive tracks are not involved in the simulation model.
- The contact surface between the piezoelectric layer and the PET substrate is classified as a bounded contact, whereby they cannot move and rotate with respect to each other (neither sliding nor separating is possible).
- The contact between the PET substrate and the supports is classified as fictional contact, whereby they can slide on the top of each other but cannot be separated or rotated.

## 4. Results and Discussion

### 4.1. Optimizations of Smart Piezoelectric Coating-Based FEM

#### 4.1.1. Influence of Radius Ratio

**Figure 7:**
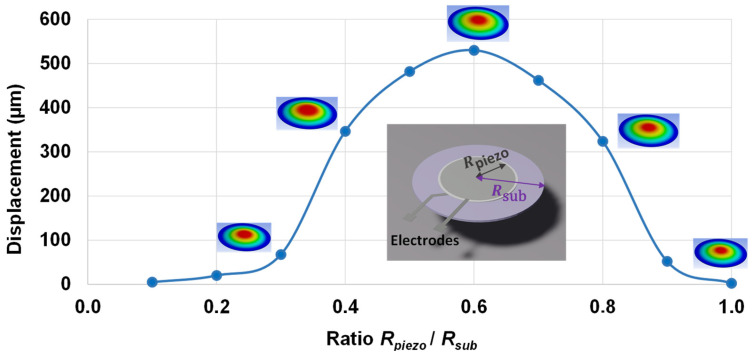
Optimization of the radius ratio between the coating and the substrate to obtain maximal displacement for a circular actuator.

This study investigates the effect of the radius dimension on the performance of a piezoelectric wafer actuator, i.e., consisting of a piezoelectric layer coated on a structural membrane substrate, as displayed in the inset of Figure 7. The simulation model relied on a dynamic finite element model (FEM), previously described in Section 3.2. The optimum ratio between the radius of the piezoelectric layer and that of the substrate was determined to maximize the wafer displacement along the thickness direction. The displacement was simulated with a given AC voltage of 50 V/µm amplitude and under the first resonance frequency, while the radius ratio between the active element and the substrate was varied from 0.2 to 1. It was revealed that the resonance frequency was close to 200 Hz, which was almost unchanged independent of the radius ratio. As seen in Figure 7, a ratio of ~0.6 offered the greatest displacement of the wafer actuator. This finding is somehow coherent with that reported in the study of Poncet et al. [83], in which the piezoelectric stack (comprising a 4.7 µm PVDF–TrFE ferroelectric polymer film sandwiched between 800 nm thick PEDOT–PSS bottom and top electrodes) was manufactured out of polymer substrates such as polyethylene naphthalate (PEN).

The result of Figure 7 was definitively considered for further device development, wherein the haptic button consisted of a 30 mm diameter PET substrate. Consequently, the piezoelectric diameter was chosen to be equal to 18 mm so as to achieve the best actuator performance.

#### 4.1.2. Influence of Thickness Ratio

Similarly, thickness optimization has been revealed to be crucial in the design of piezoelectric devices. A finite element model (FEM) was also exploited to understand the influence of the ratio between the active composite thickness (denoted $t_{piezo})$ and the PET substrate’s one (denoted $t_{sub})$ on the wafer’s displacement. 

To simplify the model and reduce the computational time, the two thin electrodes were discarded from the model. The FEM, thus, only consisted of a PET substrate and a BaTiO_3_ composite with two different thickness of 28 μm or 56 μm. The one with 28 µm corresponded to a single-layered piezo-actuator (as the thickness of each layer is typically around 25–30 µm), while the one with 56 µm was dedicated to a two-layered sample. The screen printing technique allows for an elaboration of very thin film layers, which is otherwise a veritable challenge in the case of conventional methods [73,84,85]. On the one hand, reducing the thickness to <25–30 µm for each piezoelectric layer is feasible but time-consuming (even with screen printing technique), since additional processing steps such as etching or polishing are needed. On the other hand, increasing the number of active layers could lead to higher thickness and displacement. However, the whole device might be more rigid because of additional electrode layers, which in turn affects the actuation ability itself. This explains why the number of the piezoelectric layers was limited to two in this study.

**Figure 8:**
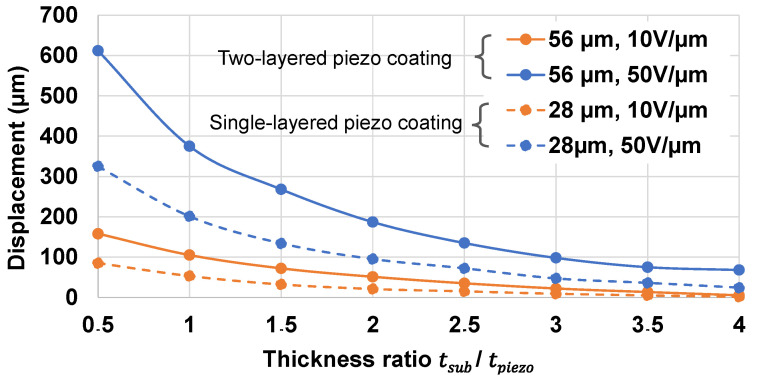
Optimization of the thickness ratio between the coating and the substrate to obtain maximal displacement for circular actuators including one-layered (dashed lines) or two-layered (solid lines) piezoelectric coating. Each sample was subjected to an electric field of 10 V/µm (orange) or 50 V/µm (blue).

The thickness of the PET model was varied in such a way that the thickness ratio $t_{sub}/t_{piezo}$ was within the interval [0.5; 4]. To assess the actuation performance, each sample was excited by a constant electric field (denoted E) of 10 V/μm or 50 V/μm. Figure 8 shows how the displacement of the wafer changes as a function of the thickness ratio. For a given input electric field, a lower ratio gave rise to higher displacement, thus improving the haptic perception. A ratio of 0.5 resulted in the best displacement, but would weaken the structure (too thin substrate) and reduce the durability of the device [86]. Therefore, it is important to consider both haptic performance and structural strength when optimizing the thickness of the piezoelectric layer. From the practical point of view, a ratio of 1.5 was revealed to be an appropriate choice to achieve the best compromise regarding performance, durability, and user comfort. For instance, the single-layered piezoelectric button designed with $t_{piezo}=28\;\mu m$ and $t_{sub}=50\;\mu m$ (i.e., the standard size of PET available in the market) allowed obtaining the optimized thickness ratio. In the case of two-layered samples (56 µm thick), the displacement was boosted to almost double as opposed to the single-layered ones, which is coherent due to their double thickness. Indeed, for the multilayered structure, the piezoelectric layers behaved as capacitors parallelly connected together; thus, they were all powered under the same electric field. Considering that these capacitors were identical in terms of piezoelectric sensitivity (the same *d*_33_ coefficient), it can be assumed that all of them resulted in the same deformation ($S_{33}$) as in the case of the one-layered sample. Hence, the displacement of the multilayered structure was boosted as a function of the number of layers (i.e., proportional to the thickness). Last but not least, the multilayer architecture allowed improving the actuation performance without changing the input voltage level. Inversely to its single-layered counterparts, a much higher voltage is needed to achieve such an actuation ability, provoking an increased probability of electrical breakdown of the device [87].

#### 4.1.3. Influence of Young’s Modulus

This study aimed to assess how the stiffness of the PET substrate affects the actuation behavior of the piezoelectric button. Usually, a rigid material will create a button capable of withstanding the force of a fingertip; however, in exchange, the sensation of touch might be deteriorated. This compromise needs to be considered in the design of the piezoelectric haptic button whose stiffness could be varied via two solutions [88]:

- Altering the PET substrate thickness ($t_{sub})$: This is an easy solution but it might not modify the thickness ratio of the structure, which was chosen to be equal to 1.5.
- Using another material of substrate with a different Young’s modulus (denoted Y): Materials with a higher Y are stiffer and more resistant to deformation under load, while those with a lower Y are more flexible and, thus, more easily stressed.

**Figure 9:**
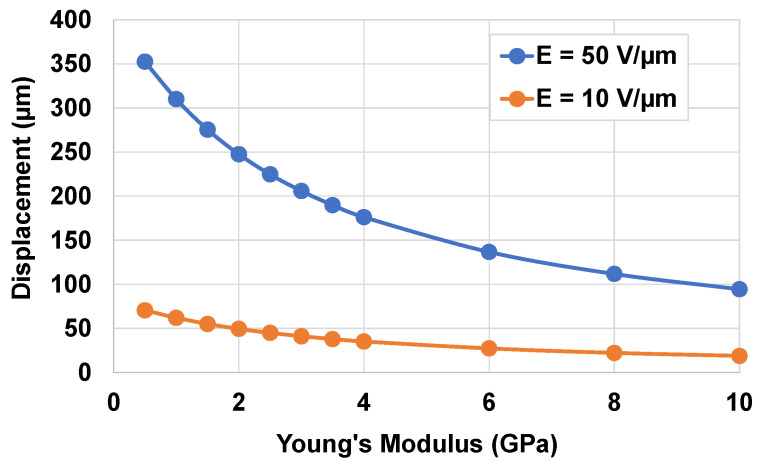
Influence of the Young’s modulus on the displacement of the single-layered actuator excited by different electric fields of 50 V/µm and 10 V/µm.

To simulate the mechanical properties of the substrate, a “fictional material” available from ANSYS library was chosen. The mass density and the Poisson coefficient of the substate were respectively set as 952 kg/m^3^ and 0.34, whereas its Young’s modulus (Y) varied within 0.5–10 GPa. The thickness of the substrate remained constant at 50 µm. Figure 9 illustrates the maximum displacement of the wafer as a function of Y under two different levels of the input electric field. Independently of the electric field, a higher Y induced a decrease in the displacement. The chosen PET with its low Young’s modulus of 2.6 GPa led to sufficient displacement to perform satisfactory human perception (e.g., ~50 µm under a low input electric field of 10 V/µm). The stiffness of the button could be somewhat adjusted by modifying the thickness of the substrate. Accordingly, this study confirmed the good mechanical characteristics of the PET to match the specifications of the haptic system and device stiffness.

#### 4.1.4. Influence of Electrodes

In the previous FEMs, the electrodes were not intentionally considered so as to reduce the computational time of simulation, as well as skip their effect with respect to other factors. From the practical point of view, however, the presence of electrodes is mandatory for applying the electric field across the piezoelectric layer, which generates the mechanical deformation and resulting haptic feedback. This study aimed to analyze the impact of electrodes on the actuation behavior of the piezoelectric wafer. In general, the electrodes should be as thin as possible to minimize their influence on the mechanical properties of the device [38]. However, they must also be good conductors and durable to withstand an important number of deformation cycles. Common materials used for electrodes in piezoelectric devices include gold, platinum, aluminum, and silver [89]. In our case, silver was chosen due to its excellent electrical conductivity, good adhesion properties, and adaptability to 3D printing additive manufacturing (AM). 

**Figure 10:**
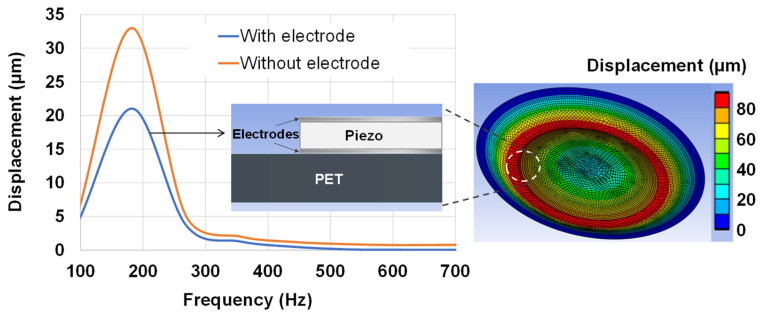
Frequency response of the displacement-based FE model of single-layered piezoelectric specimens (with or without electrode) subjected to an electric field of 10 V/µm at 200 Hz.

To better emphasize the influence of the electrodes, two different FEMs (with and without electrodes) were created via ANSYS. Both models consisted of a PET substrate and BaTiO_3_ piezoelectric layer with identical characteristics. The “harmonic response” block function was used to observe the maximum displacement as a function of the frequency within a range of 100–700 Hz. As revealed in Figure 10, the full piezoelectric wafer (with electrode) resulted in a decrease in displacement by approximately 25–30% as opposed to the simplified one (without electrode). This can be explained by the fact that the electrodes, with their non-negligible thickness (around 5–10 µm), were stiffer than the piezoelectric material and, thus, limited its ability to deform. In other words, the electrodes created a mechanical constraint on the piezoelectric layer, reducing its motion when subjected to a given input voltage. It is interesting to note that the resonance frequency for the first reflection mode (~180 Hz) was not impacted by the presence of the electrodes. 

In spite of the decrease in the actuation ability, the full piezoelectric wafer still achieved sufficient displacement (beyond the detection threshold of 2 µm, i.e., the minimum penetration for the user to feel something [19]) at frequencies below 300 Hz. Beyond that frequency, the displacement may not have reached the desired value, especially in the presence of electrode. Reducing the electrode thickness could improve the displacement, but too thin electrodes might be fragile and easily deteriorated by a considerable number of cycles of mechanical solicitation. An alternative solution involves the development of multilayered piezoelectric actuator. Indeed, when multiple piezoelectric layers are stacked together, they can generate a larger displacement due to the increase in the total electric charge across the thickness of the piezoelectric layers. This phenomenon is known as the “stacking effect” [90]. The stacking effect can be further enhanced by arranging the polarity of the piezoelectric layers in a certain pattern. For example, if the piezoelectric layers are arranged with alternating polarities, the overall displacement of the button can be increased. This is known as the “poling pattern” or “polarization pattern” [90]. However, adding more piezoelectric layers can also increase the stiffness of the button, which may affect the haptic performance. Therefore, it is important to find a balance between the number of layers and the desired displacement. The simulation results indicated that two stacked piezoelectric layers were enough to achieve the desired displacement.

The two-layered piezoelectric model was built via ANSYS, in which the contact between the two layers was assumed to be bonded. In other words, there was no slipping liaison between them, and they behaved as a single entity. The piezoelectric layers were arranged with alternating polarities, meaning that the surfaces in contact with the two adjacent piezoelectric layers had opposite polarities. If the top layer had a positive polarity, then the layer beneath had a negative polarity. This arrangement created an electric field opposite in direction between adjacent layers, resulting in an increase in the overall displacement of the button when a voltage was applied. This is because the electric field generated by the top layer was reinforced by the electric field generated by the layer beneath it, resulting in a larger net displacement of the button.

**Figure 11:**
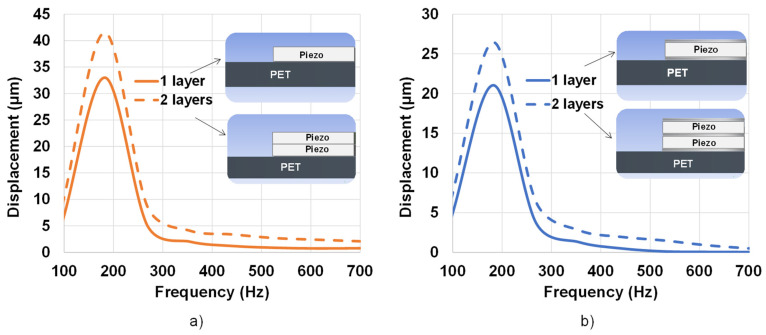
Frequency response of the displacement of single- and two-layered coating samples subjected to an electric field of 10 V/µm using FE model: (**a**) without electrode; (**b**) with electrode.

Figure 11a displays the frequency response of the one-layered and two-layered samples without electrodes. It can be seen that increasing the number of layers indeed enhanced the displacement value, especially around the resonance frequency. Such an increase may not have been as much as expected (less than double) but it enabled the button to reach the desired limit displacement of 2 µm within the bandwidth of 100–700 Hz. Samples with the electrodes achieved similar enhancement using the two-layered design, but with smaller displacement. Satisfactory haptic perception could be attained with frequencies less than 500 Hz. To ensure sufficient vibration feeling for users above 500 Hz, a higher input electric field was necessary to boost the displacement above the detection threshold, e.g., 20 V/µm instead of 10 V/µm. This result allowed validating the architecture of the two-layered piezoelectric button, which was subsequently implemented in the experimental prototype described below. 

### 4.2. Performance of Haptic Device Based on Experimental Results

**Figure 12:**
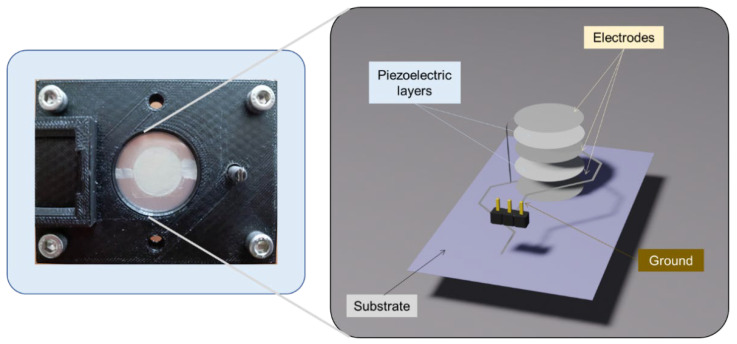
Wafer structure of a haptic button implemented on a homemade support together with the 3D design of a two-layer piezoelectric composite coated on a PET substrate.

This study focused on the assessment of actuator and sensor performances of the developed haptic button using experimental characterization setups (described in Section 3.1). Figure 12 illustrates the circular piezoelectric film clamped to the edge by a customized support. The 3D image on the right-hand side indicates the electrical connection of the electrodes to create a parallel capacitor-like structure of the two-layered piezoelectric coating.

#### 4.2.1. Displacement Spectrum

This experimental setup (described in Section 3.1.1) was conducted with the aim of validating the above simulation results, as well as measuring the actual displacement of the piezoelectric specimen when subjected to a moderate AC electric field of 10 V/μm. In particular, it was essential to verify whether or not the device could generate a feedback displacement with amplitude superior to 2 μm. For a given frequency of the applied voltage, the velocity of the piezoelectric specimen was acquired, and the maximum displacement (denoted $d_{max}$, also defined as the amplitude of displacement) could be inferred according to Equation (3).

**Figure 13:**
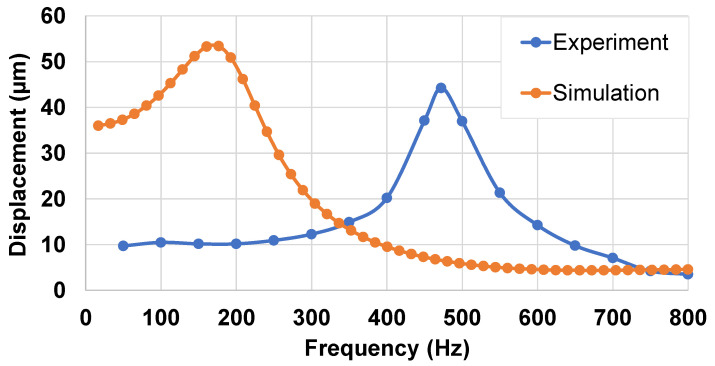
Displacement spectrum (without load) determined on the basis of experiment and simulation.

Figure 13 displays the experimental and simulation curves of the displacement spectrum, which induced the resonance frequency for the first reflection mode ($denoted\;f_{r1}$), respectively equal to 470 Hz and 180 Hz. The experiment led to a significant discrepancy of $f_{r1}$ with respect to the numerical model. This could be attributed to the following factors:

- In the simulation, the outer radius of the wafer was considered constant as the wafer was supposed to be fixed (i.e., static condition). Inversely to reality, the wafer could move slightly when pressed by a fingertip. In other words, measurements were conducted in a quasi-static environment, which is not similar to the case of the simulation.
- There was a discrepancy between the Young’s modulus of materials (BaTiO_3_ composite and PET substrate) specified in ANSYS and of the real ones.
- In practice, the wafer may not have been centered exactly on the support, producing some small changes in its geometry and, thus, shifting the resonance frequency to some extent.
- The imperfections in the fabrication process led to variations in the thickness and properties of the different layers.

In spite of the discrepancy in the resonance frequency, the experimental and numerical curves led to a displacement beyond the haptic detection threshold (2 μm) within a bandwidth of 100–700 Hz. This means that, if the user puts their finger on the wafer without applying pressure (F≈0 N), they can detect some kind of vibrations. To improve the user perception, the level of these vibrations can be changed by simply adjusting the applied electric field. 

#### 4.2.2. Actuator Mode: Displacement Versus Force 

This section investigates the displacement behavior as a function of the input pressing force exerted by a customized artificial finger. Although the target application required the developed button capable of withstanding a pressing force not greater than 5 N, experiments were carried out by varying the force in a larger range of 0–12 N. After detecting the force exerted by the finger through a generation of an electrical signal (sensor mode), actuator mode was activated. During such a configuration, a constant electric field with adjustable amplitude of 10–60 V/µm at a fixed frequency of 300 Hz was applied to the piezoelectric wafer, which in turn induced the mechanical vibrations perceived by the finger skin. The aim here was to determine the adequate levels of the applied electric field (denoted E) to conduct the displacement of the button beyond the detection threshold (~2 µm) of human perception. 

**Figure 14:**
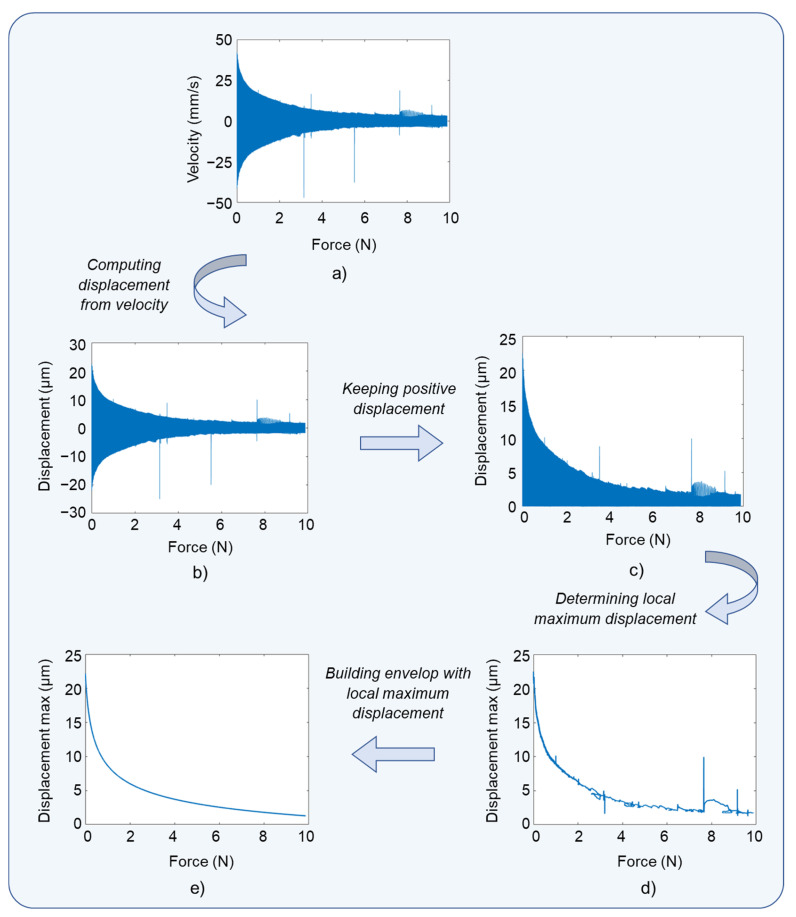
Steps used to calculate envelope of the maximum displacement as a function of the input force pressed by an artificial finger: (**a**) raw data of the velocity; (**b**) displacement computed from the velocity; (**c**) displacement remined with positive values only; (**d**) use of “local maximum function” to determine the local maximum of the displacement; (**e**) envelope of the maximum displacement based on interpolation method. The actuation test was performed under an applied electric field of 60 V/µm at a frequency of 300 Hz.

As illustrated in Figure 14a,b, the displacement was computed from the velocity signal captured by the laser vibrometer. The details of the experimental setup were addressed in Section 3.1.2. To facilitate assessment of the actuation performance, post-treated data based on the maximum value of the displacement (see Equation (3)) was employed instead of real-time data, which allowed discarding the influence of undesired electrical noises. As shown in Figure 14c,d, a MATLAB (version R2020b) algorithm was developed to compute the local maximum displacement ($d_{max})$. The identification of $d_{max}$ was then interpolated to obtain a smooth envelope, as suggested in Figure 14e. The data treatment shown as an example in Figure 14 corresponded to the wafer subjected to an electric field of 60 V/µm. 

**Figure 15:**
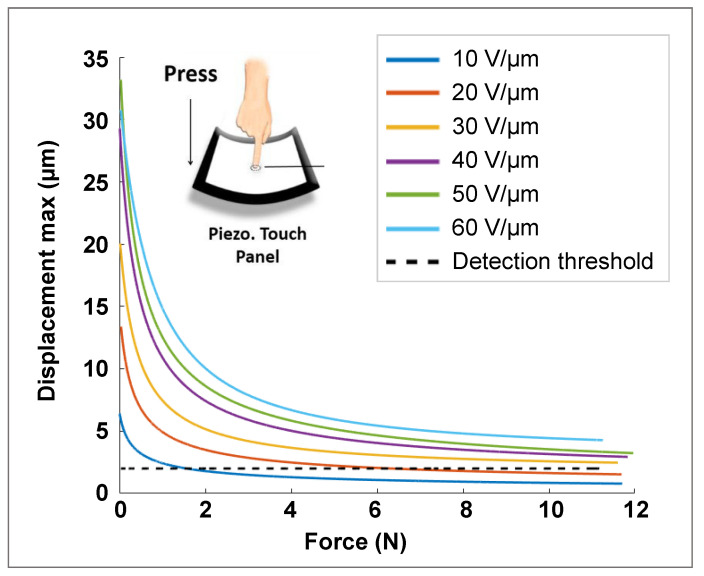
Displacement versus load of the piezoelectric specimen when subjected to different levels of electric field from 10 to 60 V/µm.

Figure 15 summarizes all the results of the treatment with the electric field (E) varied from 10 to 60 V/µm. Whatever the level of E, $d_{max}$ induced by the piezoelectric button drastically dropped even with the apparition of a small force (from 0 N to 2 N). Above 2 N, however, $d_{max}$ gradually decreased with an increase in the input force. In the case of E≥30 V/µm, $d_{max}$ was revealed to be beyond the detection threshold in the full range of force (0–12 N). Usually, the normal touch of a fingertip does not exceed 5 N; thus, a lower value of E as 20 V/μm is expected to be enough to meet the requirements. Such a value is clearly an adequate choice to get the best compromise between haptic performance and breakdown probability of the piezoelectric composite, which would be dramatically deteriorated during high-electric-field application.

#### 4.2.3. Sensor Mode: Activation of Haptic Response

Our objective in sensor mode is to detect the user input when the button is pressed, and to determine when and how a haptic response is sent to the user. Therefore, this stage, characterizing the transition from the sensor mode to the actuator mode, is of crucial consequence. Here, we distinguish the ways of pressing the haptic button: “quick”, “medium”, and “long” presses, related to durations of 0.2 s, 0.5 s, and 1 s, respectively. 

**Figure 16:**
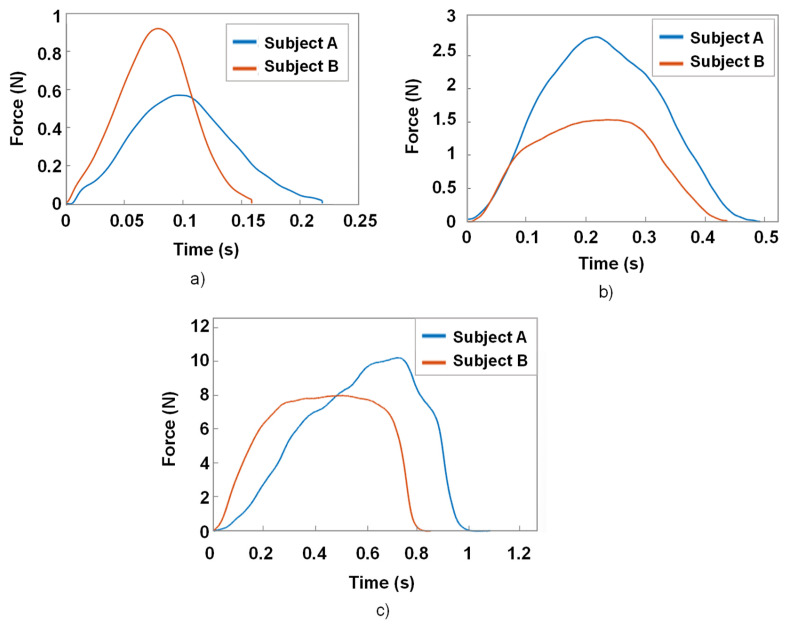
Time evolution of the external pressing force under three configurations: (**a**) quick press; (**b**) medium press; (**c**) long press. Tests were conducted on two different subjects.

Figure 16 displays the time evolution of the force measurement based on those three types of press performed by two subjects. Although multiple presses were recorded for each subject, only one of them was shown. Experiments allowed demonstrating the curve’s shape by keeping the maximum force and press duration consistent. As observed, the force increased quickly from 0 N to the maximum value (denoted $F_{max}$), and then decreased at a similar rate. The value of $F_{max}$ strongly depended on the press duration, as well as the subject. Logically, a longer duration led to a higher force value. Whatever the type of press, the forces driven by both subjects could be somewhat described by a quadratic function (or a second-degree polynomial) in which only one peak was present. After several trials, it was revealed that the medium and short durations of around 0.2–0.5 s corresponded to natural gestures of a finger when pressing a tactile button. In these cases, the pressing force did not exceed 3 N, confirming the reliability of the specifications, in which $F_{max}$ could not exceed 5 N. Regarding the long duration of 1 s, the force could exceed 10 N. This explains why, in the previous study (cee Figure 15), the range of the input force was extended to 12 N. Indeed, a long press could exist in reality, but only on some occasions for some user purposes. Accordingly, this type of press was not considered among the most used when describing the natural gestures for a touchscreen interface. 

**Figure 17:**
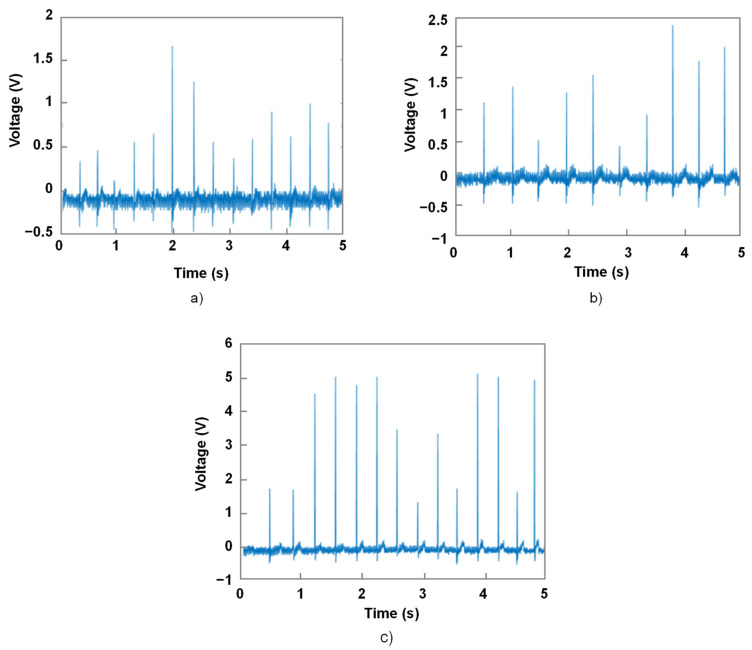
Piezoelectric response via the output voltage measured under three configurations: (**a**) quick press; (**b**) medium press; (**c**) long press.

Figure 17 illustrates the piezoelectric sensor response corresponding to different types of button presses. Here, we focused particularly on the medium presses, as they were supposedly the most commonly used in everyday scenarios. To achieve this, the voltage level deduced from the data of Figure 17b was determined. The level of this detection point was selected equal to 0.5 V, which was much higher than the electrical noise level that could cause false detections. The detection point could also be set at 0.3 V to detect quick presses as well, but this would make the button somehow more sensitive to mechanical vibrations, as well as electrical noises, resulting in unwanted haptic responses. Lastly, determining the voltage detection level for a mean press made the design of a haptic feedback system more reliable, allowing for more accurate detection of the user input with less sensitivity to noises and vibrations.

The piezoelectric sensing performance of BaTiO_3_-based devices, made with the same material and process as those conducted in this work, was determined in our recent research [38,76]. On the basis of the experimental characterizations, it was pointed out that the sensor exhibited a perfectly linear relationship between the output electrical signal and the input mechanical solicitation. The piezoelectric sensitivity of the BaTiO_3_ ink used in our studies was found to be equal to approximately 1.3. This value is somewhat small compared to common piezoelectric composites reported in the literature, which was certainly due to the low concentration of BaTiO_3_ in the composite ink. In our previous research, we investigated different solutions to boost the piezoelectric coefficient of BaTiO_3_ composites, such as aligning the particles within the polymer matrix using dielectrophoresis at the early curing stage, adding an adequate surfactant into the matrix to enhance the dispersion homogeneity, or increasing the filler content [91,92]. Optimizations of the material and process to achieve piezoelectric ink adequate for 3D printing, together with improvements in system design, are considered the key objectives to open new perspectives for higher-performance haptic devices.

### 4.3. Development of Haptic Demonstrator

#### 4.3.1. Description of Prototype

The final goal of this research was to develop a haptic demonstrator that could produce multiple sensations for people to experience. An interactive device was designed and prototyped, with the aim of being visually appealing and user-friendly. The demonstrator comprised two haptic buttons that could be programmed to generate a distinct sensation. Since the prototype was set to have one waveform generator and one amplifier, only one button could be activated at a time in actuator mode.

When a button is pressed, the corresponding piezoelectric element starts vibrating, and two LEDs light up. After the haptic response ends, there is a short delay, which can be configured before the piezoelectric voltage returns to zero. A yellow LED indicates when it is possible to press a button again. A Python program controls all the electronics and allows the user to change the input waveform applied to each button through a graphical user interface (GUI). The haptic button demonstrator not only provides an opportunity for users to experience different sensations but also allows for customization and control of the stimuli. The GUI interface is a user-friendly way to modify the waveform and adjust the parameters of the haptic response. This feature enables the user to tailor the haptic feedback to their liking and experiment with different combinations to produce unique sensations.

**Figure 18:**
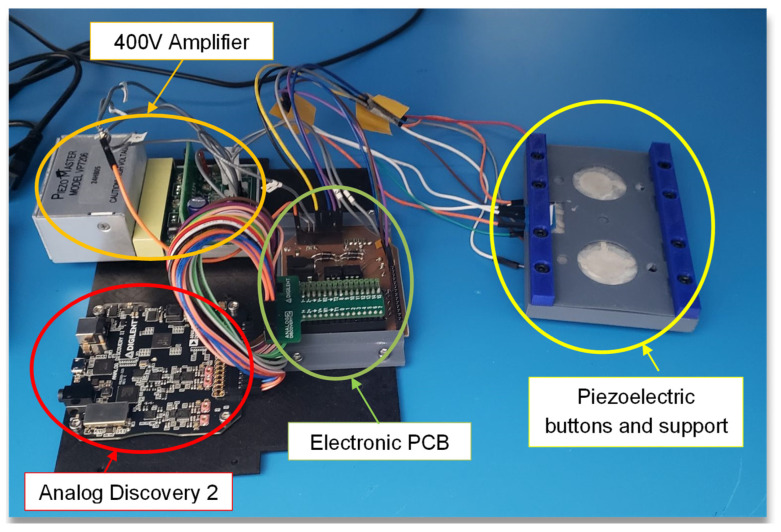
Prototype of haptic device including electronic cards and two piezoelectric buttons.

Finally, the haptic demonstrator device was successfully developed, as presented in Figure 18, consisting of two piezoelectric buttons and their support, a printed circuit board (PCB), a 400 V amplifier PiezoMaster, and the Analog Discovery 2 (AD2) device. 

#### 4.3.2. Preliminary Trials

The haptic demonstrator provided three types of signals: sinusoidal, square, and triangular. According to the theory, vibrations around 300 Hz supposedly obtain the best human perception, although those within the range of 100–700 Hz could be detected by the device. During the testing phase, it was found that some signals generate undesirable audible sounds coming from the piezoelectric buttons. In order to classify the perception and comfort as a function of the input waveform signal, two categories were investigated with respect to vibration sensibility and audible sound:

- Haptic perception: How do users perceive the vibrations?
- Audible sound: How loud are the sounds coming from the button?

We conducted multiple trials on signals with a fixed frequency of 300 Hz, as well as a sweep within a frequency range of 100–700 Hz. Tests were carried out on 20 subjects who had to respond to the above questions by expressing their satisfaction with the perception and sound according to the classification of Table 3. The degree of satisfaction can be expressed by the quantitative number from 0 % (not satisfied at all) to 100 % (totally satisfied), corresponding to the qualitative symbol from (− − −) to (+ + +). The results obtained from the tests are compiled in Table 4, reflecting the average response of the 20 subjects. In the experiments, the sinusoidal signal with a sweep was the most enjoyable sensation because a majority of subjects could feel the vibrations very clearly without any unwanted noise. The square wave was also a good signal, but the triangular wave was not effective as vibrations were difficult to detect and produced uncomfortable noises. It seems that the performance with respect to haptic quality and audio sound was somehow related. For instance, in the triangular wave, the haptic perception was poor, potentially due to the fact that an important aspect of mechanical energy was transformed into acoustical energy, leading to small vibration and undesired sound. This behavior, to some extent, was inverse in the case of the sine wave consisting of only one harmonic.

It is worth noting that there was a non-negligible dispersion of the 20 subjects when testing the haptic demonstrator. Actually, people had different preferences for the types of signals they enjoyed. Some individuals perceived the square wave vibrations to be better than the sinusoidal ones. Additionally, some participants did not like the sweep option but preferred signals around 300 Hz instead. It is clear that there was no particular type of signal that was preferred by everyone, as people had varying preferences for sensation of touch. 

**Figure 19:**
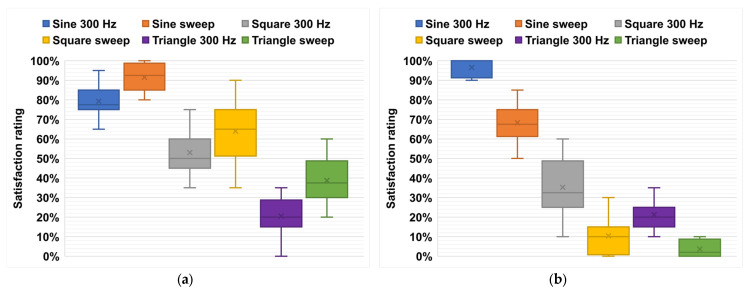
Whisker–box graphs describing the satisfaction rating of 20 subjects tested with the haptic device using different waveforms as feedback vibrations: (**a**) satisfaction with the perception; (**b**) satisfaction with the audible sound.

Figure 19a,b show the boxplots representing a statistical overview of the results (comprising locality, dispersion, and skewness) acquired from the 20 subjects who were asked about their satisfaction with the haptic perception and the acoustical sound. Table 5 summarizes the numerical indicators consisting of the mean value, the standard deviation (SD), and the maximum and minimum values of the satisfaction rating within the interval 0–100%. The three quartiles are also reported, where $Q_{1}$ is the 25th percentile (also called the lower quartile), $Q_{2}$ is the 50th percentile (i.e., the median of the entire dataset), $Q_{3}$ is the 75th percentile (also called the upper quartile), and IQR is the interquartile range. To better assess the variability of the data, we provide here an estimation of the quartile coefficient of dispersion (QCD, given by Equation (6)). A higher QCD value denotes greater dispersion of the data.

(6) $$QCD=\frac{Q_{3}-Q_{1}}{Q_{3}+Q_{1}}=\frac{IQR}{Q_{3}+Q_{1}}.$$

The detailed data related to the box graph given in Table 5 lead to the following conclusions:

- Concerning the haptic perception, the sine wave was revealed to be the most preferred by the subjects as opposed to the triangle one, regardless of which frequency was selected for the waveform (fixed at 300 Hz or swept in a range of 100–700 Hz).
- Concerning the audible sound, most subjects found that all the waveforms within the sweep led to more undesirable acoustical noises than those with the fixed frequency (300 Hz). Particularly in the case of the square and triangular signals, the noises became extremely insupportable because of their decomposition into multiples of the fundamental harmonic, in the audible high-frequency range [67].
- By combining the audible and tactile sensations, the sine wave at 300 Hz was demonstrated to be the most appropriate choice in the generation of feedback vibrations (see Figure 19).
- In all cases, the data distributions were almost symmetrical, as the medians (horizonal lines in the whisker box) were close to the mean values (green cross); thus, the skewness was near to zero.
- No observations showed any outliers or extremes values (i.e., falling below $Q_{1}-1.5\;IQR$ or above *Q*_3_ + 1.5 *IQR*), meaning that the highest and lowest occurring values were within this limit interval.
- Finer analysis regarding the data dispersion via QCD led to the following conclusions:
  ✓ For both perception and audible tests, the dispersion of the data was relatively low in the case of the sine wave, as most subjects appreciated this configuration.
  ✓ Inversely, very high variation was observed with the other waveforms, especially in the audible tests. Indeed, the QCD value was found to be equal to 74% and 100% for the square and triangle signals, respectively. This is due to the fact that more than 25% the population expressed their absolute unsatisfaction vis-à-vis the acoustical noises (i.e., $Q_{1}\approx 0)$.

Lastly, a haptic device is a complex system in which the developed material must be capable of operating in both sensor and actuator configurations. The most challenging issues relate to the fact that the system behavior strongly depends on the action and sensitivity of the human, which are supposedly variable and even unpredictable to some extent. Designing a system able to adapt to a large range of the input forces applied by users, as well as their variable perception, is considered of primary significance to improve the performance of the haptic feedback device.

Concretely, for the actuator mode, changing the input force might affect the actuation ability of the haptic system. In particular, a high force level can drastically reduce the displacement generated by the wafer, which in turn declines the perception of the user or, in the worst case, attenuates it entirely. In this case, a higher voltage must be applied to the piezoelectric wafer to ensure displacement beyond the detection threshold of human skin. Increasing the input voltage, nonetheless, also increases the probability of the electrical breakdown of the composite. Different solutions can be envisaged such as the following:

- Improving (or changing) the chemical formulation of the polymer composite so that it can support a higher electrical breakdown level. This solution is somehow time-consuming as several tests need to be conducted to verify whether or not the new matrix exhibits suitable flexibility and is compatible with AM (involving factors such as Young’s modulus, viscosity, adhesion with the substate, and UV-curability).
- Reducing the coating’s thickness, as well as that of the substrate, to maintain an optimized ratio. The decrease in sample thickness, however, should be constrained, as too thin a device might certainly weaken the structure and alter the button stiffness, in turn affecting the sensation of touch.
- Applying a variable electric field in accordance with the variation of the input force. This solution seems to be the most appropriate to achieve the best haptic performance, which should be investigated in future work, but is quite challenging, especially in the case of high-dynamic load.

On the other hand, for the sensor mode, if the input force level is too low, the output signal delivered by the piezoelectric wafer will be disturbed by electrical noises. Consequently, the trigger cannot occur, and, as a result, no feedback motion will be generated for the user, whereby they cannot feel anything. 

In summary, a human’s perception and action, which differ to a certain extent different, should be considered in the design of haptic devices. Validation of such devices with convincing results requires practical tests conducted on an important number of subjects so as to achieve reliable statistical analyses. These critical issues, together with enhancements in device development and human–machine interaction, will definitely lead the way toward the final achievement of a reliable touchscreen system.

## 5. Conclusions

This study demonstrated the high potential of piezoelectric materials in the development of haptic buttons for a tactile screen. These materials exhibit excellent ability to function as sensors for detecting user input, and as actuators for sending feedback vibration. Moreover, they are adaptable to 3D printing additive manufacturing and can be coated in thin film layers, leading to easy integration for potential applications such as in-car touchscreen interfaces. Design optimization based on FEM via ANSYS was carried out, revealing that several factors related to the geometric parameters (i.e., radius and thickness ratios) of the piezoelectric wafer, the substrate stiffness, and the electrode properties could have significant impacts on the haptic performance. Experimental characterizations, together with numerical solutions, confirmed the reliability of the haptic system, in which multilayered printed coating was preferred in order to boost the resulting displacement (i.e., beyond the detection threshold of 2 µm). It was revealed that, in addition to the material properties, the haptic perception strongly depends on the characteristics of the feedback signal, which is determined by the frequency, the displacement amplitude, and the type of waveform. As demonstrated, most users could achieve a good perception generated from the haptic button within a bandwidth of 100–700 Hz, and with a minimum movement of 2 µm. Better comfort was obtained with sine and square waves, rather than triangle waves, which led to the apparition of unwanted audio noises. 

Last but not least, a haptic demonstrator was successfully developed, allowing users to experience their sensations. An electronic circuit was fabricated according to the target specifications, and a simple application was developed to easily manipulate various types of waveforms. As different people can have different sensations with respect to the waveform they receive, practical tests should be conducted on a number of subjects (~50–100) so as to better validate the quality of the piezoelectric device. The haptic demonstrator developed in this study offers an easy solution to interact with tactile technology, paving the way for the next generation of smart multifunctional materials.

Future studies will focus on material optimization so as to boost the piezoelectric actuation ability. Such an enhancement will allow lowering the input voltage subjected to the haptic device while maintaining its deformation beyond the detection threshold of human skin. To achieve this, improvements of the process via material structuration [93,94], together with an increase in the filler concentration and the use of highly anisotropic fillers (e.g., rod or wire shape instead of spherical [69]), are envisaged. As the conception of a haptic system strongly depends on its geometry and mechanical properties, another perspective of this work involves the development of design guidelines for vibration feedback regarding changes in the dimensions and architecture of the device. In a real touchscreen where several buttons need to be implemented, it is necessary to assess the feedback intensity that can be generated by smaller devices, as well as the ability to present patterns with multiple integrated actuators. For such a system, the interaction of adjacent touch points on the flexible substrate and the dependence of the lateral size on the resonance frequency have to be considered so as to reduce the coupling effects while maintaining good haptic properties.

## Figures and Tables

**Table 1:** Different classes of materials used for haptic feedback actuators.

Actuator Technology	Electromagnetic		Electrostatic	Electroactive Polymers		Piezoelectric	
	ERM	LRA	Dielectric materials	Electrostrictive polymers	Ferroelectric polymers	Ceramic piezoelectric	0–3 piezoelectric composites
Displacement output	High	High	Low	High	High	Low	High
Force output	Low	Low	Low	Average	Average	High	High
Required input voltage	Low	Low	High	High	High	Moderate	Moderate
Frequency (dynamics)	Low	Low	High	Low	Low	High	High
Response time	~50 ms	~30 ms	~1 ms	~10 ms	~10 ms	~0.5 ms	~10 ms
Sensor mode possible?	No	No	No	yes, but pseudo mode	Yes	Yes	Yes
Flexibility	Non	Non	Yes	Yes	Yes	Non	Yes
Energy consumption	High	Average	Low	Low	Low	Low	Low
Economic cost	Moderate	Moderate	Moderate	High	High	High	Moderate

**Table 2:** Parameters of the analytical actuator model.

Material	Properties	Symbol
PET substrate		
Density	*ρ*	952 kg/m^3^
Poisson’s ratio	*v*	0.34
Young’s modulus	*Y*	2.62 GPa
Diameter	*d*	30 mm
Thickness	*t*	50 µm
Piezoelectric composite		
Density	*ρ*’	3000 kg/m^3^
Poisson’s ratio	*v*’	0.37
Young’s modulus	*Y*’	7 GPa
Diameter	*d*’	18 mm
Thickness	*t*’	28 µm
Relative permittivity	*ε*’	10.5
Piezoelectric constants	*e*_31_ = *e*_33_ *e*_15_	0.016 C/m^2^ 0 C/m^2^

**Table 3:** Degree of satisfaction (as a percentage) regarding the touch and audible sensation perceived by the humans’ fingertip.

Symbol	− − −	− −	−	~	+	+ +	+ + +
Satisfaction (%)	0–15	15–30	30–45	45–55	55–70	70–85	85–100
Perception	Nothing	Bad	Disappointed	Average	Nice	Good	Excellent
Audible sound	Unsupportable	Loud	Noisy	Ambient	Soft	Low	Quiet

**Table 4:** Degree of satisfaction (as a percentage) concerning the perception and audible sensation of human. Tests were performed with 20 subjects on the haptic demonstrator for different waveform/frequency.

Waveform/Frequency	Human Sensibility	Audio-Sound
Sine/300 Hz	+ +	+ + +
Sine sweep/100–700 Hz	+ + +	+
Square/300 Hz	~	−
Square sweep/100–700 Hz	+	− − −
Triangle/300 Hz	− −	− −
Triangle sweep/100–700 Hz	−	− − −

**Table 5:** Statistical data related to the satisfaction level with the haptic perception and the audible sound. Experimental tests were performed on 20 subjects using different types and frequencies of the output waveform to generate the feedback vibrations.

Waveform	Sine/300 Hz	Sine/Sweep 100–700 Hz	Square/300 Hz	Square/Sweep 100–700 Hz	Triangle/300 Hz	Triangle/Sweep 100–700 Hz
Satisfaction rating (%) in haptic perception						
Mean	79.25	91.5	53	64	20.5	38.75
SD	6.75	6	9.3	12.6	7.55	9.25
Min	65	80	35	35	0	20
Max	95	100	75	90	35	60
Q1	75	85	45	53.75	15	30
Q2	77.5	92.5	50	65	20	37.5
Q3	85	96.25	60	75	26.25	46.25
IQR	10	11.25	15	21.25	11.25	16.25
QCD (%)	6.25	6.21	14.29	16.50	27.27	21.31
Satisfaction rating (%) in audible sound						
Mean	96.5	68.25	35.25	10.4	21.25	3.65
SD	3.85	7.75	12.775	7.26	6.125	3.58
Min	90	50	10	0	10	0
Max	100	85	60	30	35	10
Q1	93.75	63.75	25	2.25	15	0
Q2	100	67.5	32.5	10	20	2
Q3	100	75	46.25	15	25	6.25
IQR	6.25	11.25	21.25	12.75	10	6.25
QCD (%)	3.23	8.11	29.82	73.91	25.00	100.00

## Data Availability

Not applicable.
